# Circulating microRNAs as Potential Novel Diagnostic Biomarkers to Predict Drug Resistance in Temporal Lobe Epilepsy: A Pilot Study

**DOI:** 10.3390/ijms22020702

**Published:** 2021-01-12

**Authors:** Selene De Benedittis, Francesco Fortunato, Claudia Cava, Francesca Gallivanone, Enrico Iaccino, Maria Eugenia Caligiuri, Isabella Castiglioni, Gloria Bertoli, Ida Manna, Angelo Labate, Antonio Gambardella

**Affiliations:** 1 Department of Medical and Surgical Sciences, Institute of Neurology, University “Magna Graecia”, Germaneto, 88100 Catanzaro, Italy; selene.db90@gmail.com (S.D.B.); francescfortunato@gmail.com (F.F.); labate@unicz.it (A.L.); a.gambardella@unicz.it (A.G.); 2 Institute of Molecular Bioimaging and Physiology (IBFM), National Research Council (CNR), Via F.Cervi 93, 20090 Segrate-Milan, Italy; claudia.cava@ibfm.cnr.it (C.C.); francesca.gallivanone@ibfm.cnr.it (F.G.); 3 Department of Experimental and Clinical Medicine, University “Magna Graecia” of Catanzaro, 88100 Catanzaro, Italy; iaccino@unicz.it; 4 Neuroscience Research Center, University “Magna Graecia” of Catanzaro, 88100 Catanzaro, Italy; me.caligiuri@unicz.it; 5 Department of Physics “Giuseppe Occhialini”, University of Milan-Bicocca, Piazza della Scienza 3, 20126 Milan, Italy; isabella.castiglioni@unimi.it; 6 Institute of Molecular Bioimaging and Physiology (IBFM), National Research Council (CNR), Section of Germaneto, 88100 Catanzaro, Italy

**Keywords:** temporal lobe epilepsy, miRNAs, diagnosis, prognosis, antiseizure medications, ASMs

## Abstract

MicroRNAs (miRNAs) are small noncoding RNAs that have emerged as new potential epigenetic biomarkers. Here, we evaluate the efficacy of six circulating miRNA previously described in the literature as biomarkers for the diagnosis of temporal lobe epilepsy (TLE) and/or as predictive biomarkers to antiepileptic drug response. We measured the differences in serum miRNA levels by quantitative reverse transcriptase polymerase chain reaction (qRT-PCR) assays in a cohort of 27 patients (14 women and 13 men; mean ± SD age: 43.65 ± 17.07) with TLE compared to 20 healthy controls (HC) matched for sex, age and ethnicity (11 women and 9 men; mean ± SD age: 47.5 ± 9.1). Additionally, patients were classified according to whether they had drug-responsive (*n* = 17) or drug-resistant (*n* = 10) TLE. We have investigated any correlations between miRNAs and several electroclinical parameters. Three miRNAs (miR-142, miR-146a, miR-223) were significantly upregulated in patients (expressed as average expression ± SD). In detail, miR-142 expression was 0.40 ± 0.29 vs. 0.16 ± 0.10 in TLE patients compared to HC (*t*-test, *p* < 0.01), miR-146a expression was 0.15 ± 0.11 vs. 0.07 ± 0.04 (*t*-test, *p* < 0.05), and miR-223 expression was 6.21 ± 3.65 vs. 1.23 ± 0.84 (*t*-test, *p* < 0.001). Moreover, results obtained from a logistic regression model showed the good performance of miR-142 and miR-223 in distinguishing drug-sensitive vs. drug-resistant TLE. The results of this pilot study give evidence that miRNAs are suitable targets in TLE and offer the rationale for further confirmation studies in larger epilepsy cohorts.

## 1. Introduction

Temporal lobe epilepsy (TLE) is the most common form of drug-resistant focal epilepsy, and it is considered a network disorder involving widespread structural alterations beyond the putative epileptic focus [1,2]. Hippocampal sclerosis (HS) is the most common pathological substrate of TLE [3], which can be detected by magnetic resonance imaging (MRI), but extra-hippocampal abnormalities are also frequently observed [4,5]. Incomplete knowledge of pathological changes in TLE complicates a therapeutical approach; indeed, 50–70% of patients with TLE are refractory to drug treatment with antiseizure medications (ASMs), and the presence of HS has been used to predict poor ASM response in these patients [6]. In 2010, the International League against Epilepsy (ILAE) addressed the need for a definition of drug resistance in epilepsy, declaring that “drug-resistant epilepsy may be defined as the failure of adequate trials of two tolerated and appropriately chosen and used ASM schedules (whether as monotherapies or in combination) to achieve sustained seizure freedom” [7]. Despite the increasing number of studies that have been performed over the last few decades, the exact mechanism underlying drug resistance in epilepsy remains unclear. The pathological mechanism underlying TLE may involve abnormal gene expression regulation, including post-transcriptional networks. In transcriptional process regulation and the control of epigenetic gene expression, microRNAs (miRNAs) play an important role. miRNAs are small noncoding RNAs that span between 19 and 24 nucleotide bases. They gain biological activity through base pairing in the 3′-untranslated regions of target messenger RNA (mRNA) molecules, thereby guiding a protein complex termed the RNA-induced silencing complex (RISC). Binding of the RISC to the mRNA sequence results in either the inhibition of translational processes or the degradation of the mRNA of its target gene [8]. Each miRNA can regulate one or more target genes, while several miRNAs can regulate the same target gene. miRNAs regulate the expression level of proteins in cells through this complicated network [9]. Dysregulated miRNA expression has been associated with inflammatory pathways, cell death, neuronal excitability, and synaptic reorganization, which underlie epileptogenesis (the gradual process by which a normal brain changes to an epileptic brain) [10,11]. Increasing knowledge about the function of miRNAs makes them candidates of interest as biomarkers of diseases and in the management of therapy [12]. In addition, miRNAs can be detected in blood and serum, making them suitable candidates as potential circulating biomarkers to assess disease risk and treatment responses. The identification of circulating biomarkers for resistant epilepsy could potentially improve the choice of correct treatment as well as the prognosis of epileptic patients. Indeed, many circulating miRNAs have been reported as differentially expressed in hippocampi and peripheral blood from both animal models and patients with temporal lobe epilepsy compared to nonepileptic subjects [13,14,15,16]. Considering that few studies have specifically examined correlations between miRNA and TLE, the objectives of this study are (i) to determine if changes in expression of six epilepsy-related miRNAs, namely, miR-146a-5p, miR-142-5p, miR-132-3p, miR-138-5p, miR-298, and miR-223-3p (from now on, they are named miR-146a, miR-142, miR-132, miR-138, miR-298, miR-223), are present in the serum of a TLE cohort (these miRNAs have been selected on the findings of a previous work [11]), and (ii) to verify if the serum levels of these miRNAs are potentially associated with failure of ASMs. Indeed, to perform this part of the study, we selected TLE patients who showed no response to at least two different ASM therapies. Lastly, the identification of mechanisms coordinating gene networks in patients with TLE will help to identify novel therapeutic targets and biomarkers.

## 2. Results

### 2.1. Characterization of Selected Patients

A total of 47 participants (including 20 healthy subjects and 27 TLE divided into 17 drug-sensitive and 10 drug-resistant TLE patients) were recruited to this study. All patients were on mono or polytherapy ASMs at their last clinical visit. Inclusion and exclusion criteria are described in the Material and Methods section. The detailed clinical characteristics of individuals are listed in Table 1. No significant differences in age or gender were found (all *p* > 0.05). 

### 2.2. miR-142, miR-146a, and miR-223 are Possible Diagnostic Circulating Molecules

**Figure 1:**
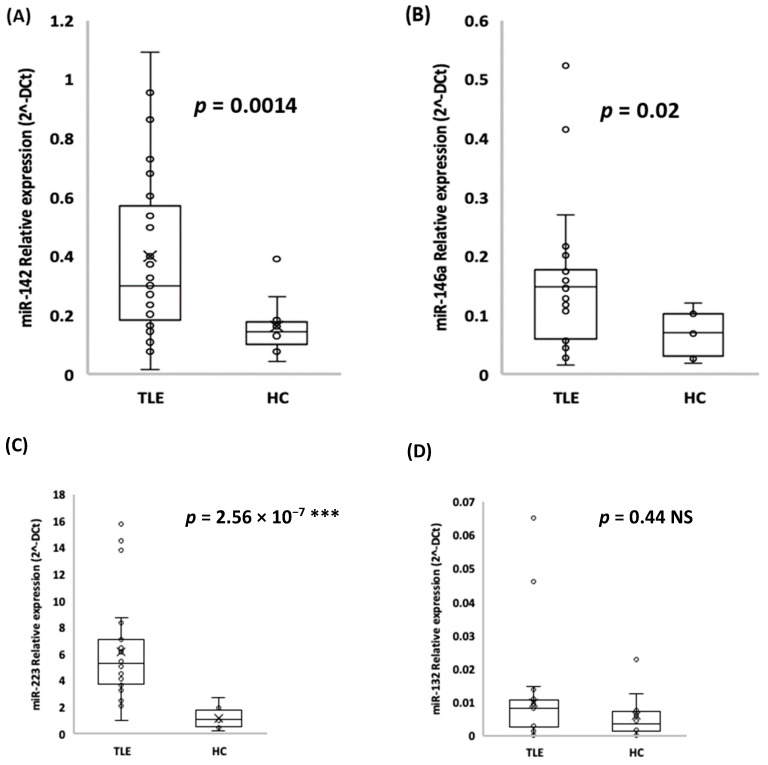
miR-142 (**A**), miR-146a (**B**), miR-223 (**C**), and miR-132 (**D**) relative expression in the TLE patients compared to healthy control (HC) subjects (average of expression ± standard deviation (SD); *t*-test, *** *p*-value < 0.001).

Two miRNAs were excluded from subsequent analyses, namely, miR-138-5p and miR-298, as they did not show any significant change in their expression. The relative expression analysis in qRT-PCR revealed that miR-142, miR-146a, and miR-223 were significantly upregulated in the TLE patients compared to the HC subjects (Figure 1A–C). In particular, miR-142 average expression ± SD was 0.40 ± 0.29 vs. 0.16 ± 0.10 in the TLE patients compared to the HC subjects, respectively (*t*-test, *p*-value < 0.01); miR-146a average expression ± SD was 0.15 ± 0.11 vs. 0.07 ± 0.04 in the TLE patients compared to the HC subjects, respectively (*t*-test, *p*-value < 0.05); miR-223 average expression ± SD was 6.21 ± 3.65 vs. 1.23 ± 0.84 in the TLE patients compared to the HC subjects, respectively (*t*-test, *p*-value < 0.001). Additionally, miR-132 was upregulated in sera from the TLE patients compared to the HC subjects, but the difference was not significant (average expression ± SD was 0.01 ± 0.014 vs. 0.006 ± 0.007, respectively; t-test, p-value >0.05; Figure 1D).

### 2.3. miR-142 and miR-223 are Possible Prognostic Molecules for Drug-Resistant Subjects

**Figure 2:**
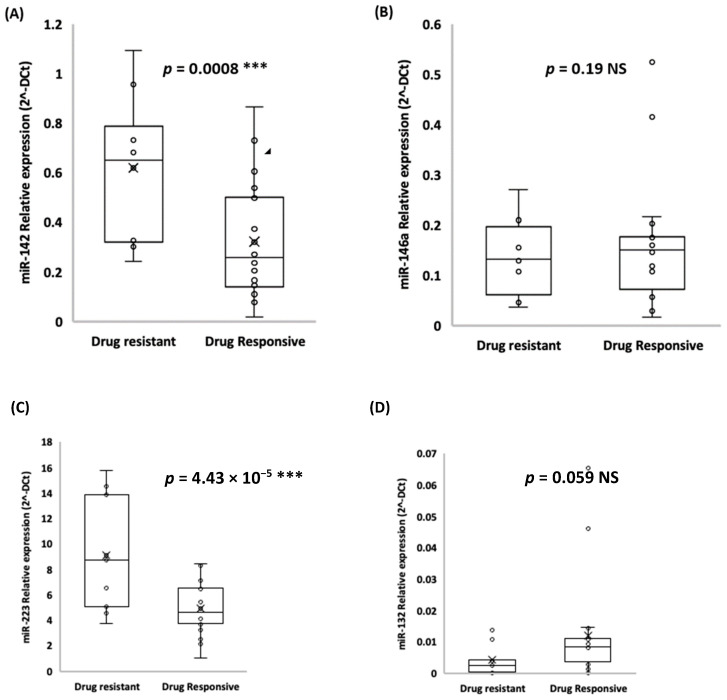
miR-142 (**A**), miR-146a (**B**), miR-223 (**C**), and miR-132 (**D**) relative expression (average expression ± SD) in drug-sensitive versus drug-resistant TLE patients (*t*-test, *** *p*-value < 0.001).

Relative expression analysis in qRT-PCR revealed that miR-142 and miR-223 were significantly upregulated in drug-resistant patients compared to drug-sensitive patients. In particular, miR-142 average expression ± SD was 0.62 ± 0.34 vs. 0.32 ± 0.24 (*t*-test, *p*-value < 0.001) and miR-223 average expression ± SD was 9.14 ± 4.80 vs. 4.97 ± 2.11 (*t*-test, *p*-value < 0.001) in drug-resistant patients compared to drug-sensitive patients, respectively (Figure 2A,C). Regarding the other two miRNAs, we found that both miR-146a and miR-132 were both downregulated in drug-resistant TLE sera, but the difference with drug-sensitive TLE subjects was not significant (Figure 2B,D).

### 2.4. Correlation Analysis of miRNA Expression

**Figure 3:**
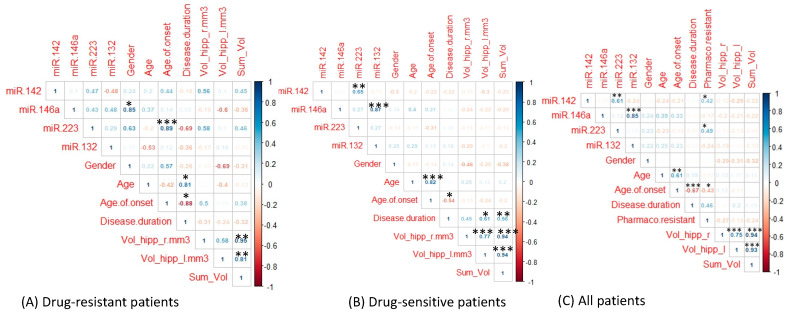
Correlation matrix in (**A**) drug-resistant patients, (**B**) drug-responsive patients, and (**C**) all patients. Positive correlations are visualized in blue color and negative correlations in red color. Color intensity of the text labels is proportional to the correlation coefficients. Significant p-values corresponding to the correlation coefficient are indicated with asterisk (* *p*-value < 0.05; ** *p*-value < 0.01; *** *p*-value < 0.001).

The correlation coefficients between miRNA expression and several parameters in drug-resistant and drug-sensitive patients were calculated (Figure 3). In drug-resistant subjects, the analysis revealed a significant correlation between miR-146a and gender (*p*-value < 0.05) and miR-223 and age of onset (*p*-value < 0.001). In the same group, we found a correlation trend between some miRNAs and imaging features, although without a significant p-value. In particular, miR-142 and miR-223 and right hippocampal volume showed a correlation coefficient of 0.56 and 0.58, respectively; miR-146a and left hippocampal volume showed a negative correlation coefficient of −0.6 (Figure 3A). The lack of significance was possibly due to the reduced numerosity of the population. 

In the drug-sensitive group, we found a significant, positive association between miR-142 and miR-223 (*p*-value < 0.01), miR-146a and miR-132 (*p*-value < 0.001), age and age of onset (*p*-value < 0.001), disease duration and left hippocampal volume (*p*-value < 0.05), and right hippocampal volume and left hippocampal volume (*p*-value < 0.001) (Figure 3B). We also calculated the correlation coefficients in all patients, including drug-resistant patients and drug-sensitive patients, and we found a significant, positive association between miR-142 and miR-223 (*p*-value < 0.01), miR-146a and miR-132 (*p*-value < 0.001), miR-142 and pharmaco-resistance (p-value <0.05), and miR-223 and pharmaco-resistance (*p*-value < 0.05) (Figure 3C). Finally, our correlation studies suggested a positive correlation between age and disease duration (*p*-value < 0.05) in drug-resistant patients and a negative correlation between age of onset and disease duration (*p*-value < 0.05) (Figure 3A). In the drug-sensitive group, there was a significant negative correlation between age of onset and disease duration (*p*-value < 0.05; Figure 3B). These results are in line with similar published findings [17,18].

### 2.5. Prognostic miR-223 and miR-142 are Involved in the Control of Inflammation and Phagocytosis Processes

**Figure 4:**
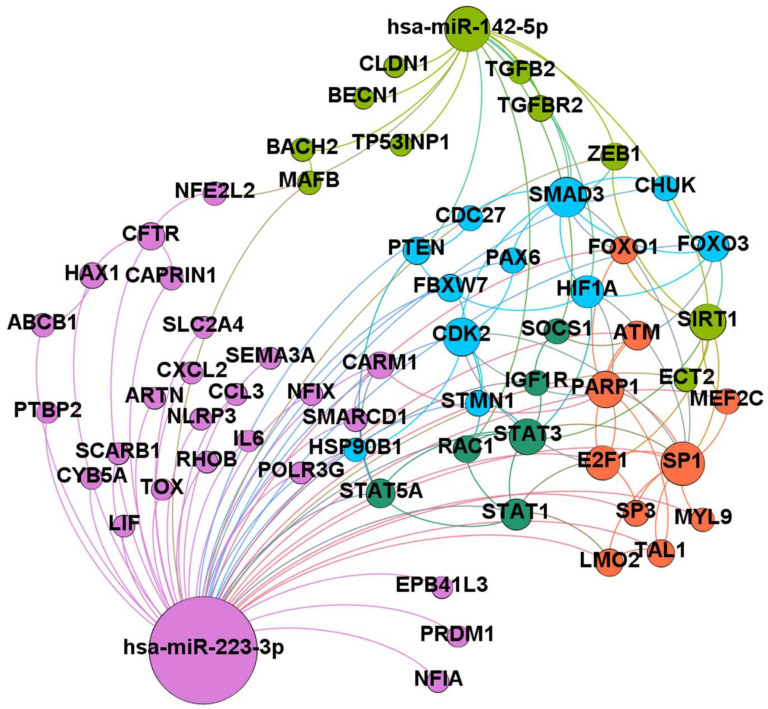
miRNA–protein network. The overall connectivity of miR-223-3p and miR-142-5p was examined by assembling miRNA–mRNA associations and protein–protein interactions. The size of the nodes is proportional to degree centrality. The five modules found are colored in purple, blue, orange, light-green, and dark-green color.

**Figure 5:**
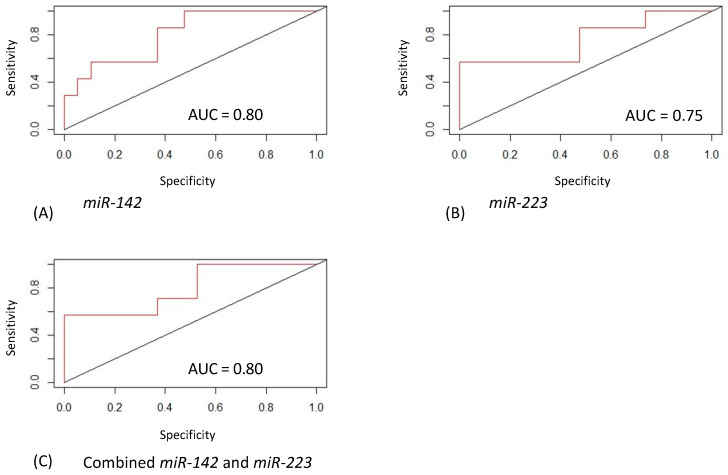
Classification performance by receiver operating characteristic (ROC) to discriminate responsive vs. nonresponsive epilepsy patients. A logistic regression model was applied using expression levels of (**A**) miR-142 (area under curve (AUC): 0.80), (**B**) miR-223 (AUC: 0.75), and (**C**) combined miR-142 and miR-223 (AUC: 0.80).

We constructed a miRNA–mRNA network integrating miRNA–mRNA associations and protein–protein interactions. We found a network of 134 interactions and 94 nodes. Figure 4 represents the most connected network. From the network, we obtained 5 modules consisting of 26 (purple color), 13 (light-green color), 11 (blue color), 7 (orange color), and 6 nodes (dark green color). The nodes with a higher degree centrality were SP1 (degree centrality of 13); SMAD3 (degree centrality of 11); CDK2 (degree centrality of 10); PARP1, SIRT1, and STAT3 (degree centrality of 9); E2F1 (degree centrality of 8); HIF1A (degree centrality of 7). SP1, the gene with the highest degree of centrality, is a direct target of miR-223. CDK2, PARP1, STAT3, and E2F1 are direct targets of miR-223. SMAD3, SIRT1, and HIF1A are direct targets of miR-142. The results obtained from a logistic regression model showed the good performance of miR-142 and miR-223 in distinguishing drug-sensitive vs. drug-resistant epileptic patients. Receiver operating characteristic (ROC) curve analysis showed an AUC of 0.80 for miR-142 (Figure 5A), an AUC of 0.75 for miR-223 (Figure 5B), and an AUC of 0.80 for combined miRNAs (Figure 5C). AUC summarizes the ROC curve and measures the overall performance of a binary classifier. It ranges from 0.5 to 1.0, where 0.5 indicates the performance of a random classifier and 1 represents a perfect classifier. 

## 3. Discussion

### 3.1. miR-142, miR-146a, and miR-223 are Diagnostic Circulating Molecules

With the aim of identifying new circulating epilepsy biomarkers, our study focused on the miRNA extracted from the serum selected in a previous publication [11]. We isolated and analyzed the serum of epileptic patients, looking for that group of six miRNAs, namely, miR-146a, miR-142, miR-132, miR-138, miR-298, and miR-223. qRT-PCR analysis performed on patient sera showed that miR-142, miR-146, and miR-223 are possible circulating diagnostic molecules for epilepsy as it is possible to distinguish between epilepsy individuals and healthy subjects. Both miR-142 and miR-146a have been already described in several models of epilepsy [19,20]. 

Recently, miR-146a has been proposed as a diagnostic molecule in a panel of circulating miRNAs for genetically generalized epilepsy [21], possibly because this miRNA is involved in perpetuating inflammation of the hippocampus, as described in a chronic TLE rat model [22]. Moreover, in childhood forms of epilepsy, miR-146a with miR-106b has been found to be upregulated in serum of patients; the authors proposed these two miRNAs as diagnostic molecules, with a significant AUC value [23]. miR-142 and miR-223 were found to be upregulated in epileptogenic tubers from a tuberous sclerosis complex, where their expression levels were associated with inflammatory signaling [24]. To our knowledge, it is the first time that miR-223 has been proposed as a circulating diagnostic molecule in TLE.

### 3.2. miR-142 and miR-223 are Prognostic Molecules for Drug-Resistant Subjects

One of the most important challenges for epileptic pathology is the “a priori” identification of which epileptic patients will respond to ASM treatment in order to avoid unnecessary drug treatments. We thus evaluated whether diagnostic miRNAs were able to distinguish the two populations, namely, drug-sensitive from drug-resistant patients. qRT-PCR analysis showed that miR-142 and miR-223 were both increased in the sera of drug-resistant subjects. This observation reveals their potential use as prognostic circulating molecules, which could help in addressing the patients to a different treatment option. By analyzing its expression in a drug-resistant epileptic mouse model [25], miR-142 has already been proposed as a diagnostic molecule, but no one has proposed it for the discrimination of human sera of drug-resistant subjects. It is also the first time, to our knowledge, that miR-223 is proposed as an epilepsy-associated circulating molecule that is able to address the patient to the optimal treatment approach. Furthermore, we suggest that the miR-142 and miR-223 might be good biomarkers to classify drug-sensitive vs. drug-resistant epileptic patients, with an AUC of 0.80 and 0.75, respectively. We propose that these miRNAs could contribute to precision medicine in epilepsy by classifying the most responsive subset of patients.

### 3.3. miRNA Expression Correlates with Gender and Age of Onset of Epilepsy

Statistical analysis allows us to create a correlation matrix to identify the associations between clinical data, miRNA expression levels, and imaging features in drug-resistant and drug-responsive patients. We identified a significant, positive association between miR-146a overexpression and gender and miR-223 overexpression and age of onset in drug-resistant patients, miR-223 being upregulated in patients with a later age of onset. 

The importance of the miR-146a neuroprotective mechanism in epileptogenesis is well accepted, acting as a negative feedback regulator of the proinflammatory signaling pathway [26], but no publication has reported a correlation between miR-146a expression and gender. Equally, we were not able to find any publication regarding miR-223 correlation with age of onset. We hypothesize that miR-223 overexpression could influence the beginning of the pathology. In drug-sensitive patients, we obtained a significant, positive correlation between miR-142 and miR-223 and miR-146a and miR-132. miR-142 and miR-223 have been identified and are expressed together during hematopoiesis; they are able to attenuate the proliferation of hematopoietic cells [27]. Parallel miR-142 and miR-223 increased expression has also been found in methotrexate-resistant rheumatoid arthritis patients, and their decrease has been proposed as a marker of drug responsiveness [28]. However, despite these promising results, further studies must be performed in order to fully investigate the meaning of these associations.

### 3.4. Prognostic miR-223 and miR-142 are Involved in the Control of Inflammation and Phagocytosis Processes

Finally, we calculated the correlation coefficient for all patients (both drug-resistant and drug-sensitive patients), and we found a significant positive association between miR-142 and miR-223 (*p*-value < 0.01), miR-146a and miR-132 (*p*-value < 0.001), miR-142 and pharmaco-resistance (*p*-value < 0.05), and miR-223 and pharmaco-resistance (*p*-value < 0.05). In order to better understand the molecular mechanism of drug resistance, we generated a network involving the two miRNAs, miR-142 and miR-223, that have proven to be prognostic molecules for drug-resistant subjects. To understand the role of these prognostic miRNA, we analyzed by in-silico approach all the putative common target mRNAs regulated by both miR-223 and miR-142, generating a complex gene network. Eight genes of the network have a central role in the drug resistance process: SP1, SMAD3, CDK2, PARP1, SIRT1, STAT3, E2F1, and HIF1A. Using String analysis (https://string-db.org/), we looked in depth into the functions of the identified common target genes and we found that all the target genes are reported to be involved in the “positive regulation of cellular process” (Go term), mostly in the “regulation of cell differentiation”. Some of them are also reported as “cellular response to oxygen-containing compound” genes, also having a role in the “regulation of developmental process”. CDK2 and PARP genes are involved in cell cycle control and apoptosis; it is well known that epilepsy leads to the apoptosis of several neurons [29]. During the development of resistance, the nervous tissue of epileptic patients modifies the expression pattern of different genes, activating numerous transcriptional factors and modifying the structure of chromatin according to its transcription or repression [30]. For this reason, among the main targets of the two upregulated miRNAs, we find some transcription factors (STAT3, E2F1, SP1, HIF1a) and some chromatin remodeling molecules within the whole network (SMARCD1, CARM1, PTBP2). One pathway that seems to have a main role in drug-resistant epilepsy is phagocytosis. Microglia is composed by macrophages and phagocytes and has an emerging role as a mediator of drug resistance development [29]: after seizures, microglia cells are activated, becoming more mobile, changing their appearance, and releasing inflammatory factors and cytokines [29]. These inflammatory signals affect the behavior of nearby cells, promoting neuroprotection by attracting microglia to phagocyte dead cells or in long-lasting inflammation, leading to neurodegeneration and loss of neurons. It is thus expected to find some mRNAs of the autophagy process (i.e., SIRT1), a process required for the removal of debris of apoptotic cells, as well as several regulators of inflammation among the targets of miR-223-3p and miR-142-5p. Therefore, within the network, we found as possible targets of miR-223 and miR-142 some inhibitors of cytokine signaling (i.e., SOCS1), modulators of monocytes and neutrophils (i.e., CXCL2) of the inflammasome polymeric complex (i.e., NLRP3), and some factors involved in the inflammatory process (CCL3, IL6). A major role in the development of drug resistance seems to involve a group of transmembrane proteins that are able to regulate the permeability of epithelia or the exchange of ions and small molecules with the environment (i.e., Claudin1, CYB5A, CFTR). It is possible that upregulation of miR-223 and miR-142 leads to alterations in cell permeability, which, in turn, sustains the development of drug resistance. As expected, as a possible miRNA target, we also found multidrug resistance protein 1 (MDR1/ABCB1), the P-glycoprotein involved in the transport of drugs out of cells against concentration gradients, reducing the desired intracellular drug concentration [31]. It is a multispecific efflux transporter that binds a variety of ASMs. Numerous studies have shown that miRNAs can modulate drug resistance by regulating the expression of ABC membrane transporters such as ABCB1. Based on accumulating evidence, ABCB1 is significantly overexpressed in brain tissue samples resected from patients and in models of refractory epilepsy [32,33]. The use of specific antagonists of ABCB1 or downregulation of its expression has been shown to increase the concentrations of ASMs in the brain, thereby enhancing the therapeutic effects of these ASMs [34]. It is unexpected to find this mRNA as a potential target of two upregulated miRNAs in drug-resistant subjects since we expected tissue ABCB1 to be overexpressed in these subjects in order to obtain a reduction of the intracellular concentration of ASMs. Indeed, the overexpression of this protein, observed in the drug-resistant form of epilepsy, critically limits drug penetration into the brain by altering blood–brain barrier activity [31]. One possible explanation of our findings is that while the miRNAs that we have analyzed are in the serum, the ABCB1 target gene obtained in the in-silico analysis should be expressed by brain tissue. The difficulty in obtaining the brain tissue of drug-resistant epileptic subjects makes it impossible, at this phase, to validate the expression levels of intracellular ABCB1 mRNA. Moreover, it is possible that the development of drug resistance in epileptic patients involves several other functions of cells and the whole organism and is not only due to the increased expression of a single membrane protein (such as ABCB1).

## 4. Materials and Methods

### 4.1. Patient Recruitment

Twenty-seven consecutive adult patients of either gender with TLE were prospectively screened for study participation. Prior to undergoing any study procedures, written consent was obtained from all subjects or from their close relatives before sample collection. The study was approved by the local medical ethics committee (Protocol No. 123 on 14 May 2015) and carried out in accordance with the approved guidelines. All patients were evaluated regarding age, onset of epilepsy, history of febrile seizure, family history of epilepsy, and number of ASMs used. Data and evaluation procedures on our TLE patients have been reported in greater detail elsewhere [35]. In detail, the diagnosis of TLE was mainly based on strict criteria that are considered to be reliable indicators of TLE, according to the criteria proposed by the International League Against Epilepsy [36]. The interictal EEGs always included routine awake and sleep-deprived recordings with supplementary T1 and T2 electrodes. The localization of epileptiform abnormalities was based on the site of maximum voltage on referential montage or phase reversal on bipolar montage. EAs were diagnosed in the presence of focal spikes or sharp waves, followed by slow waves, and they always involved the temporal region as they occurred over electrodes F7, F8, T3, T4, T1, and T2. Any suggestion of seizure onset outside the mesial temporal structures by semiology or EEG findings was an exclusion criterion. Patients were excluded from this study if they were receiving nonpharmacological treatment such as Vagus Nerve Stimulation (VNS) or a ketogenic diet. All patients were examined using a 3-Tesla GE MR750 scanner (GE Healthcare, Rahway, NJ, USA). High resolution 3-Tesla cerebral MRI protocol included whole-brain, three-dimensional, T1-weighted (BRAVO), spoiled gradient recalled echo (TE/TR = 3.7/ 9.2 ms, flip angle 12°, voxel size = 1 × 1 × 1 mm^3^). Image processing was performed using FMRIB Software Library (FSL) [37], as follows: Left and right hippocampal volumes were automatically segmented using FSL-FIRST [38] and normalized for subject head sizes using the scaling factor estimated with FSL’s tool SIENAX [7] in order to reduce head-size-related variability between subjects. Results of the segmentation were visually checked by an expert and manually refined if needed. Patients were excluded if they had (1) an MRI-visible lesion (structural etiologies, including stroke, trauma, and malformations of cortical development), (2) multifocal seizure onset, or (3) dual pathology or tumors. Patients were subsequently divided into two groups according to their response to AED treatment and seizure frequency: (1) drug-responsive epilepsy, defined as seizure freedom for at least 24 months, and (2) drug-resistant TLE, based on the ILAE criteria for drug-resistant epilepsy as seizures persisting despite trials of two or three adequately tolerated and appropriate antiepileptic drugs [7]. Matched controls were Caucasian, unrelated individuals with no neurological or psychiatric disease and no history of seizures, who voluntarily agreed to donate serum samples to our study. A complete description of the 47 subjects’ characteristics is provided in Table 2.

### 4.2. Serum Processing 

Up to 6 mL of whole blood was collected from each participant and processed for serum isolation within 3 hours of collection by centrifugation at 3000 r.p.m. for 10 min at 4 °C. The serum samples were stored at −80 °C and were not thawed until use. Hemolytic serum samples were excluded.

### 4.3. RNA Extraction

Total RNA was extracted with the use of a mirVana RNA isolation kit (Thermo Fisher, Waltham, MA, USA) according to the manufacturer’s instructions. Briefly, serum samples were thawed on ice, and 0.4 mL of serum was diluted with an equal volume of mirVana PARIS 2X denaturing solution; subsequently, a synthetic spike-in control RNA (Cel-miR-39-3p), used as an internal reference control, was added to the serum. Equal volumes of acid/phenol/chloroform were added to each aliquot, and samples were subsequently centrifuged for 5 min at 10,000 × *g*. After passage through a mirVana PARIS column, several washing steps were carried out, following the manufacturer’s protocol. Finally, RNA was recovered in 100 μL of elution buffer. RNA concentration was determined by measuring a 1 μL aliquot on a NanoDrop ND-1000 spectrophotometer (Thermo Fisher) and stored at −80 °C

### 4.4. RNA Reverse-Transcription

For cDNA synthesis, the TaqMan Advanced MicroRNA cDNA Synthesis Kit (Thermo-Fisher) was used according to the manufacturer’s instructions (2 μL input volume). TaqMan probe-based qRT-PCR (Thermo-Fisher) was performed.

### 4.5. Real-Time Quantitative PCR (qRT-PCR)

TaqMan qPCR was performed using Advanced MicroRNA Assays (reaction volume 10 μL) and TaqMan Universal PCR Master Mix on reaction plates (MicroAmp Plate Optical 96-well, Thermo Fisher) and run on a QuantStudio 7 Flex Real-Time PCR System (Thermo Fisher). TaqMan Advanced MicroRNA Assay probes are available at http://www.mirbase.org/ and http://www.thermofisher.com/ and listed in Table 3.

### 4.6. MicroRNA Quantification Using qRT-PCR

All samples and no-template controls for each assay were run in duplicate. Cycle quantification values (Cq) were excluded from analysis if they did not meet the quality control criteria in QuantStudio 7 Flex Software v.2.1 (Thermo Fisher) or if they were identified as an outlier. To obtain serum levels of the miRNAs of interest, the ΔCt approach (2−ΔCt algorithm) was used. The results are presented as the average of relative values ± SD, calculated as 2 − (Ct of miRNA of interest − Ct of normalizer). From the available approaches for normalization, exogenous reference cel-miR-39 was used as a normalizer for circulating miRNAs [39,40]. 

### 4.7. Statistics

ΔCt was compared between the groups using an unpaired, two-tailed Student’s *t*-test. The significance is indicated by the *p*-value calculation. We performed a correlation analysis using the characteristics of TLE patients, including clinical data (gender, age, age of onset, and disease duration), miRNA expression levels, and imaging features. For each pair of features, we calculated Pearson’s correlation. Considering the corresponding *p*-values of the correlation, the features were considered significantly correlated if *p*-values were <0.05. A heatmap was performed using an R package using corrplot [41].

### 4.8. Bioinformatics Analysis

In order to investigate the molecular mechanisms of drug resistance in TLE, we explored mRNA targets of miR-223 and miR-142. We identified validated mRNA targets and constructed protein–protein interactions involving miRNAs and their mRNA targets using the SpidermiR R package [42]. The network was visualized using Gephi 0.9.2 [43]. We used modularity (community detection) to study the network structure. The network was divided into subnetworks based on resulting connections since subnetworks have dense interactions between the nodes within modules but sparse connections between the nodes in distinct modules. The ability of miRNAs to distinguish drug-sensitive vs. drug-resistant TLE patients was assessed by a logistic regression model. We plotted the receiver operating characteristic (ROC) curve and calculated the area under the curve (AUC). The AUC value expresses the performance of classification of a binary predictor, considering both specificity and sensitivity values. Statistical analysis was performed with the R package [44].

## 5. Conclusions

In summary, we found upregulated miRNAs in the serum of patients with TLE, in particular, three circulating possible diagnostic miRNAs (miR-142-5p, miR-146a-5p, and miR-223-3p) and two novel possible prognostic miRNAs (miR-142-5p and miR-223-3p) associated with drug resistance. Prognostic miRNA activity controls several genes, many of which are involved in cell proliferation, apoptosis, neuroinflammation, cell permeability, and autophagy. However, the exact mechanisms underlying pharmaco-resistance in TLE and how they are regulated are still unclear. Understanding the role of these miRNAs in the drug-resistant group, using a full analysis of mRNA expression, would help us in the description of the pathogenesis of drug-resistant epilepsy.

## Figures and Tables

**Table 1:** Clinical data of temporal lobe epilepsy (TLE) patients are shown (*nd*, not determinable).

Sample Name	Gender	Age (Years)	Age of Onset (Years)	Disease Duration (Years)	Pharmaco-Resistant	Vol Hipp Right mm^3^	Vol Hipp Left mm^3^
Epy 615	M	37	3	34	Yes	3200.31	3909.27
Epy 652	F	63	1	62	Yes	3455.74	3403.25
Epy 654	M	67	8	59	Yes	*nd*	*nd*
Epy 655	M	58	57	1	No	3547.00	3279.00
Epy 656	F	21	1	20	No	4349.42	4153.40
Epy 661	F	43	9	34	Yes	2644.52	3018.02
Epy 677	M	47	1	46	Yes	4035.11	3983.11
Epy 678	F	40	38	2	Yes	4701.02	3761.01
Epy 684	F	80	75	5	No	3799.00	3260.00
Epy 686	F	55	53	2	No	3101.64	3581.09
Epy 701	M	31	5	26	Yes	3615.99	3897.99
Epy 711	F	45	39	6	No	3441.01	2986.01
Epy 712	M	24	22	2	No	4028.64	3289.11
Epy 715	F	53	11	42	No	4040.45	4017.45
Epy 722	M	73	*nd*	*nd*	No	4825.33	3986.12
Epy 725	M	68	62	6	No	4411.59	4242.06
Epy 732	F	43	23	20	No	3034.62	3958.66
Epy 744	F	27	26	1	Yes	3351.00	3760.00
Epy 746	M	57	20	37	No	5673.10	5768.69
Epy 748	M	56	45	11	No	4231.99	4470.99
Epy 758	F	47	40	7	Yes	4540.00	4200.00
Epy 768	F	24	20	4	No	3615.99	3897.99
Epy 777	M	23	19	4	No	3724.39	3687.90
Epy 779	M	31	19	12	No	4094.99	3811.99
Epy 781	F	27	9	18	No	3442.62	3449.62
Epy 782	F	49	46	3	No	*nd*	*nd*
Epy 785	F	31	18	13	Yes	4070.56	4136.06

**Table 2:** Baseline characteristics of epilepsy patients (*na,* not analyzed; t-test p-values are indicated).

Serum Samples	Control (*n* = 20)	TLE (*n* = 27)	*p*-Value
Age, mean ± SD (years)	47.5 ± 9.1	43.65 ± 17.07	*p > 0.05*
Male, *n* (%)	9 (45.0%)	13 (48.1%)	*p > 0.05*
Age of onset, mean ± SD (years)	*na*	24.39 ± 20.76	
Disease duration, mean ± SD (years)	*na*	18.21 ± 17.86	
**Etiology**			
Drug-responsive, *n* (%)		17 (62.97%)	
Drug-resistant, *n* (%)		10 (37.03%)	

**Table 3:** Indication of the sequences of the candidate microRNAs and the endogenous normalizer microRNA used.

miR Base ID	NCBI Accession Number Gender	TaqMan Advanced miRNA Assay (ID)	Sequence of the Mature miRNA 5′------------------------3′
*Hsa-miR-146a-5p* (miR-146a)	MI0000477	478399_mir	UGAGAACUGAAUUCCAUGGGUU
*Hsa-miR-142-5p* (miR-142)	MI0000458	477911_mir	CAUAAAGUAGAAAGCACUACU
*Hsa-miR-223-3p* (miR-223)	MI0000300	477983_mir	UGUCAGUUUGUCAAAUACCCCA
*Hsa-miR-132-3p* (miR-132)	MI0000449	477900_mir	UAACAGUCUACAGCCAUGGUCG
*Hsa-miR-138-5p* (miR-138)	MI0000476	477905_mir	AGCUGGUGUUGUGAAUCAGGCCG
*Hsa-miR-298* (miR-298)	MI0005523	478430_mir	AGCAGAAGCAGGGAGGUUCUCCCA
